# Application of near-infrared fluorescence imaging in the accurate assessment of surgical margins during breast-conserving surgery

**DOI:** 10.1186/s12957-022-02827-4

**Published:** 2022-11-09

**Authors:** Yabing Wang, Wei Jiao, Zhaocai Yin, Wanjun Zhao, Kai Zhao, Yong Zhou, Rui Fang, Bingbin Dong, Bin Chen, Zheng Wang

**Affiliations:** Department of Thyroid and Breast Surgery, Yijishan Hospital of Wannan Medical College, Zheshan West Road No. 2, Wuhu, 241001 Anhui Province China

**Keywords:** Near-infrared fluorescence, Breast cancer, Indocyanine green, Surgical margin

## Abstract

**Objective:**

To evaluate the feasibility and accuracy of near-infrared fluorescence imaging technology for assessing margins during breast-conserving surgery for breast cancer.

**Methods:**

Forty-three breast cancer patients who received surgical treatment at Yijishan Hospital of Wannan Medical College were selected. Before the operation, the patients were administered with an indocyanine green injection of 0.5 mg/kg intravenously 2 h before operation. During and after the operation, all patients underwent surgical margin monitoring with the near-infrared fluorescence imaging system for fluorescence imaging and acquisition of images and quantitative fluorescence intensity. During the operation, the patients’ tissue specimens were collected on the upper, lower, inner, outer, apical, and basal sides of the fluorescence boundary of the isolated lesions for pathological examination.

**Results:**

Fluorescence was detected in the primary tumor in all patients. The average fluorescence intensities of tumor tissue, peritumoral tissue, and normal tissue were 219.41 ± 32.81, 143.35 ± 17.37, and 105.77 ± 17.79 arbitrary units, respectively (*P* < 0.05, *t* test). The signal-to-background ratio of tumor to peritumor tissue and normal tissue was 1.54 ± 0.20 and 2.14 ± 0.60, respectively (*P* < 0.05, *t* test). Abnormal indocyanine green fluorescence was detected in 11.6% patients (5/43), including 3 patients with residual infiltrating carcinoma and 2 patients with adenosis with ductal dilatation.

**Conclusion:**

This study confirms the high sensitivity and specificity of near-infrared fluorescence imaging technology for breast-conserving surgery margin assessment. Near-infrared fluorescence imaging technology can be used as an intraoperative diagnosis and treatment tool to accurately determine the surgical margin and is of important guiding value in breast-conserving surgery for breast cancer.

**Supplementary Information:**

The online version contains supplementary material available at 10.1186/s12957-022-02827-4.

## Background

Breast cancer is one of the most common malignant tumors in women. Recent studies have shown that its incidence has been increasing yearly [1]. Breast-conserving surgery (BCS) has become the first choice of treatment for early breast cancer. BCS results in the same survival rate as mastectomy with a better cosmetic effect. An important factor influencing recurrence after mastectomy is the surgical margin [2–4]. Histopathological examination is the optimal approach used to assess the state of the surgical margin. In traditional breast-conserving surgery, the operation surgical margin is determined mainly by the operator’s vision and touch. Reliance on these subjective factors can easily lead to the following shortcomings: (1) a high positive rate of surgical margins, (2) excessive removal of healthy breast tissue, and (3) postoperative breast deformation. To address the limitations of existing techniques, more effective perioperative strategies are needed to quickly and accurately assess the marginal state of breast cancer specimens in real time.

Indocyanine green (ICG) has been used in many medical fields, and it is the only near-infrared fluorescence (NIF) imaging reagent approved for clinical use by the China Food and Drug Administration (CFDA) and US Food and Drug Administration (FDA) [5]. The penetration depth of near-infrared light in tissue is extensive and is less affected by the background of biological tissue. Because ICG possesses near-infrared absorption and emission fluorescence properties, infrared light can be detected using a fluorescence imaging system and displayed through imaging equipment. After intravenous injection, ICG binds to serum protein and functions as a macromolecule in circulation, which is reflected in its passive enrichment in tumor tissue through the enhanced permeability and retention effect (EPR) [6, 7]. Given its high EPR in solid tumor tissue and high accumulation in tumor cells, ICG is used for tumor imaging. NIF imaging technology provides high real-time resolution and sensitivity and has been extensively investigated in recent years given its remarkable imaging performance [8]. Currently, NIF imaging based on ICG is widely applied in various surgical operations, such as liver cancer, gastrointestinal tumors, lung cancer, oral and maxillofacial tumors, breast cancer, and sentinel lymph node biopsy [9–12].

This study aimed to evaluate the accuracy of NIF imaging in the diagnosis of breast-conserving surgery. The effect of intraoperative tumor fluorescence imaging combined with postoperative histopathological examination was explored.

## Data collection and processing

### Source of cases

A total of 43 patients with breast cancer were selected from December 2020 to December 2021 in the Department of Thyroid and Breast Surgery, Yijishan Hospital of Wannan Medical College with an average age of 56.27 years. All the patients received ultrasound, mammography, and magnetic resonance imaging to determine the size of the tumor. All the patients received breast-conserving surgery and sentinel lymph node biopsy，and they were informed of their risks and signed an informed consent form.

### Criteria for inclusion

1. Patients diagnosed with primary breast cancer before surgery;
2. No signs of skin chest wall involvement;
3. Small ratio of tumor volume to breast volume;
4. Complete clinical imaging and pathological data;
5. Patients willing to conserve the breast who are in good physical condition, with no cardiopulmonary and other key diseases and can objectively and conditionally accept radiotherapy and other follow-up treatments.

### Criteria for exclusion

1. Received systematic treatment for breast cancer;
2. Patients with a history of allergies to ICG or iodine;
3. Patients with other serious underlying diseases or second primary cancer.

### Image acquisition

**Fig. 1:**
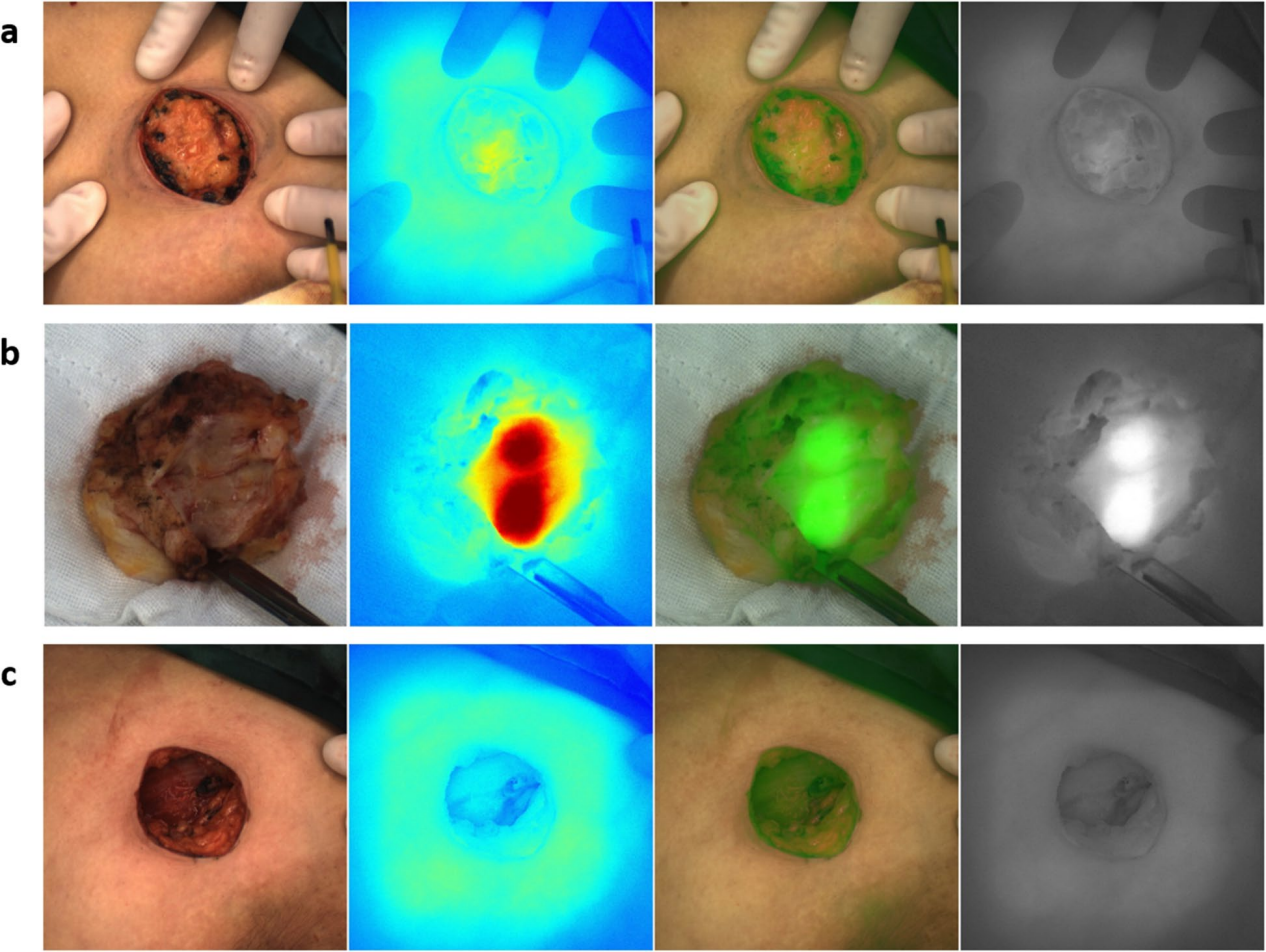
**a** During the operation, near infrared fluorescence (NIF) imaging system was used to image the tumor, and real-time navigation was applied to determine the resection edge of the tumor. **b** The distance between the tumor and surgical margin was determined, and the ratio of the tumor center to the background signal was calculated. **c** After tumor resection, the residual cavity of the breast operation was completely exposed, and NIF imaging system was used to detect fluorescence signals from the tumor bed

**Fig. 2:**
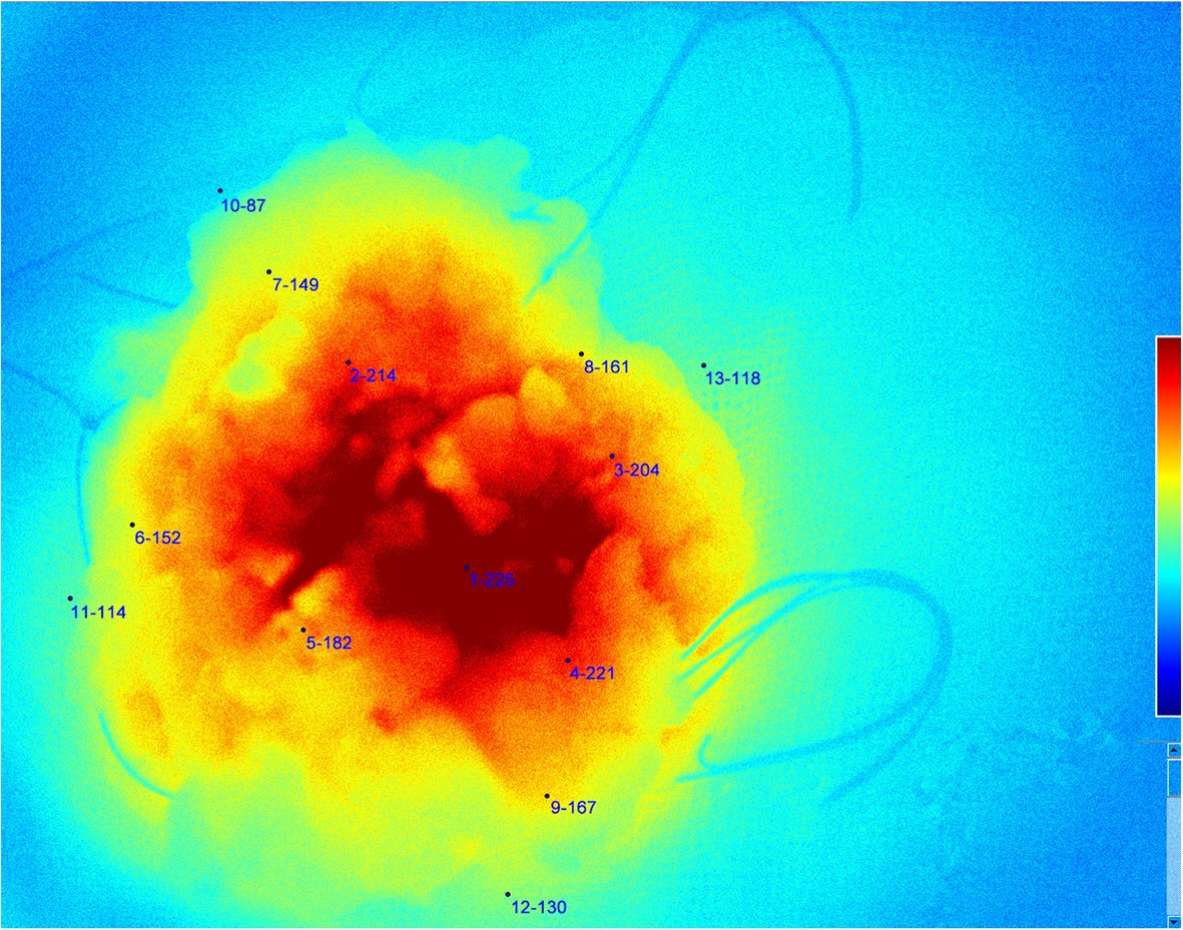
Quantitative measurement images of fluorescence intensity

**Fig. 3:**
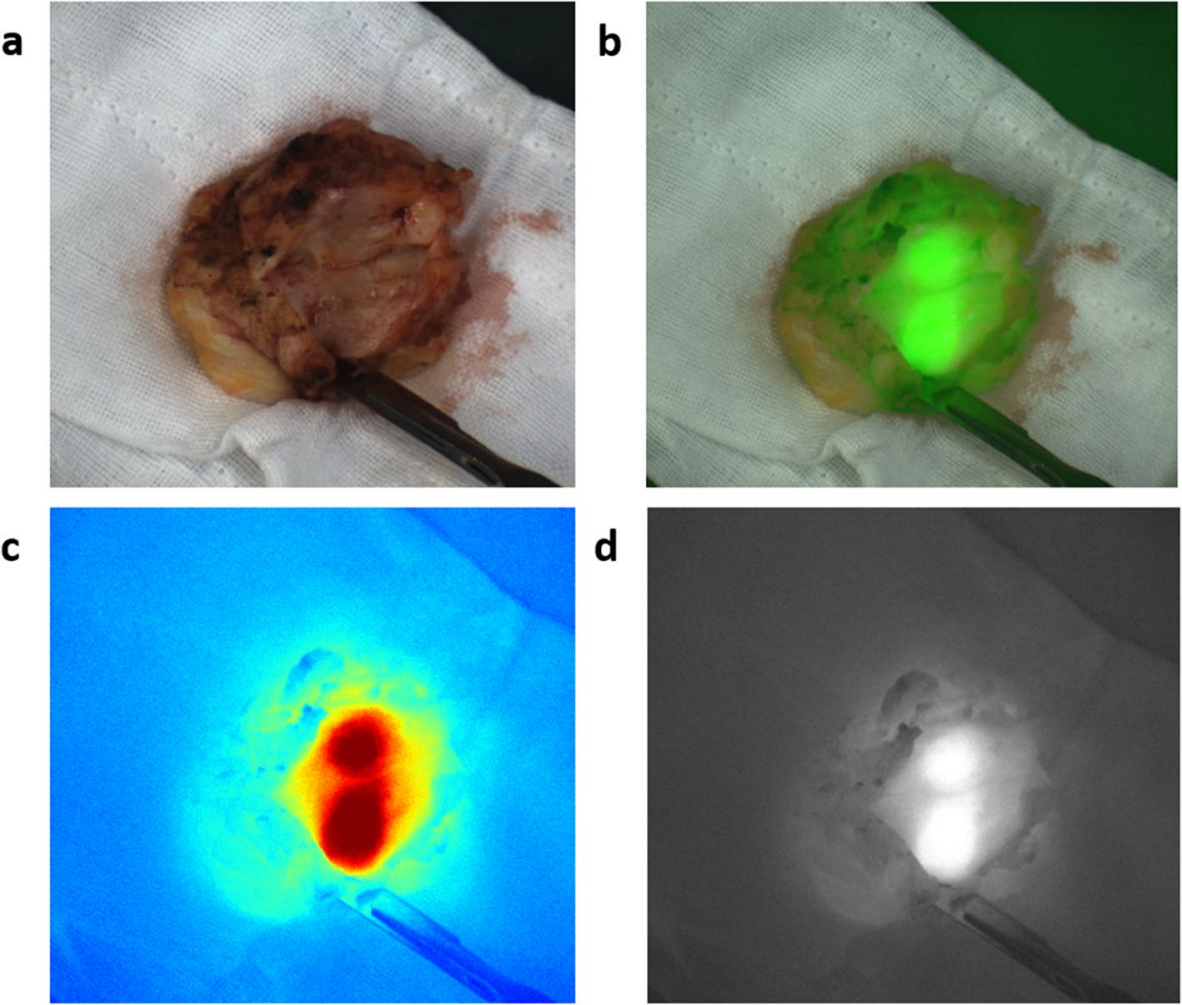
**a** Visible light image, **b** fluorescence mode image, **c** fusion image, **d** color levels image

The patients received a peripheral intravenous injection of ICG 2 h before the operation (0.5 mg/kg). Before the lesion was removed, the NIF imaging system (Real-IGS, Nuoyuan Medical Equipment Co., Ltd., Nanjing, China) was used to observe and take images of the lesion in vivo (Fig. 1a). During the operation, a NIF imaging system was used to image the tumor, and real-time navigation was applied to determine the resection edge of the tumor. Methylene blue was used to mark the preresection edge and remove the tumor along the marking line. After tumor resection, we dissected the specimen and got a tumor fluorescence imaging in vitro to assess the fluorescence intensity between tumor central and peripheral margin (Fig. 1b). The residual cavity of the breast operation was completely exposed, and the NIF imaging system was used to detect fluorescence signals from the tumor bed (Fig. 1c). In our research, abnormal fluorescence was defined as any residual tissue showing higher fluorescence intensity than what we set (136 AUS), then we performed intraoperative complementary resection to avoid any re-operation. The fluorescence of the extracted specimens was detected and recorded. The distance between the tumor and surgical margin was determined, and the ratio of the tumor center to the background signal was calculated (Fig. 2). The specimens were immediately sent to the lab for pathological examination of the state of the incisal margin. For NIF imaging, the subwindow on the display presents fluorescence images in four different modes, including visible light (Fig. 3a), color levels (Fig. 3b), fusion (Fig. 3c), and fluorescence mode images (Fig. 3d) obtained by the NIF imaging system. The fluorescence intensity was quantified using an NIF spectrometer. The measured distance was fixed at approximately 40 cm and was measured perpendicular to the tissue surface.

### Evaluation index

The signal-to-background ratio (SBR), the tumor tissue, and its surrounding tissues were identified from the excitation light images of in vivo and in vitro lesions [13]. The signal values were measured using ImageJ software, and SBR value (average gray value of tumor central tissue/gray value of peripheral margin tissue) was calculated.

### Statistical analysis

GraphPad Prism 8.0 software was used for statistical analysis. Data are expressed as the mean ± standard deviation. The SBR was used to quantify the fluorescence contrast. One-way ANOVA was used to compare the fluorescence intensity of tumor tissues, peritumor tissues, and normal tissues. *P* < 0.05 was considered to be statistically significant.

## Results

Overall, 43 surgical patients were enrolled. Patient characteristics are listed in Table 1 with an average age of 56.27 years old. Specifically, 31 patients were under 60 years old (72.09%) and 12 were over 60 years old (27.91%). In total, 29 had a tumor diameter ≤2 cm (67.44%) and 14 had a diameter of 2–4 cm (32.56%). Moreover, 35 patients (81.40%) had no axillary lymph node metastasis and 8 had axillary lymph node metastasis (18.60%) according to postoperative pathology. In addition, 16 patients had harbored luminal A tumors (37.21%), 7 had luminal B1 tumors (16.28%)，and 19 had luminal B2 tumors (44.19%). Seven patients showed HER-2 overexpression (16.28%). In total, 36 had invasive ductal carcinoma (83.72%), 2 had ductal carcinoma in situ (DCIS) (4.65%), and 5 patients had other types of carcinomas (mucinous adenocarcinoma 2，micropapillary carcinoma 3) (11.63%).

**Table 1:** Patients clinical data

Patients characteristics *(n* = 43)	Absolute no.	Relative %
Age (years)		
≤60	31	72.09%
>60	12	27.91%
Tumor size (cm)		
≤2	29	67.44%
>2	14	32.56%
Lymph node status		
No metastasis (N0)	35	81.40%
Metastasis (N1-3)	8	18.60%
Histologic type		
Invasive	36	83.72%
DCIS	2	4.65%
Others	5	11.63%
Surgical margin		
Positive	3	6.98%
Negative	40	93.02%
ER status		
Negative	8	18.60%
Positive	35	81.40%
PR status		
Negative	14	32.56%
Positive	29	67.44%
Her-2 status		
Negative	18	41.86%
Equivocal	18	41.86%
Positive	7	16.28%

**Fig. 4:**
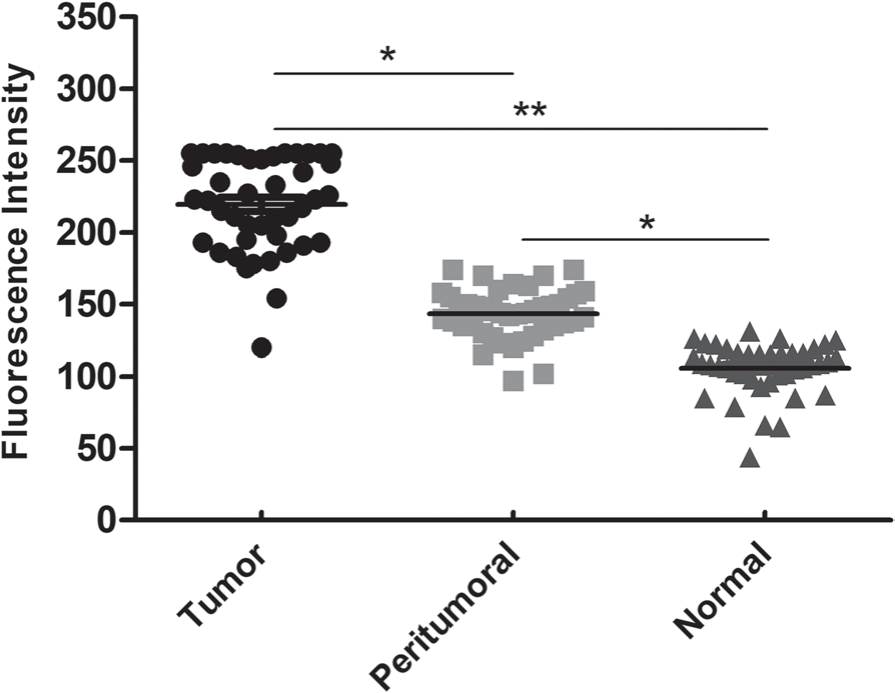
Fluorescence intensities of tumor, peritumoral, and normal tissues. *P<0.05, **P<0.01

**Fig. 5:**
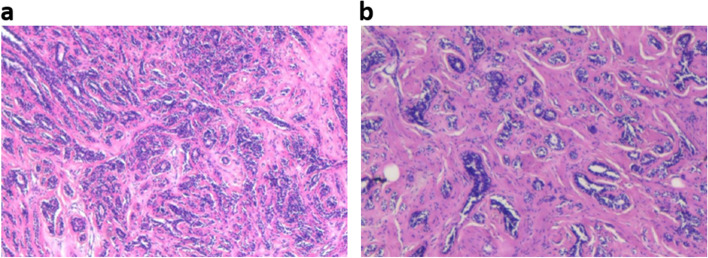
Pathological results of tissues with fluorescence developed. **a** Invasive breast cancer. **b** Breast adenosis with ductal dilatation

After ICG injection before the operation, tumor fluorescence was detected in all 43 patients. The boundary between tumor and normal tissue was clearly identified using the NIF imaging system in all cases (Fig. 2), and the fluorescence boundary was located within the surgical boundary. In this study, 93.0% (40/43) of the intraoperative imaging were clear enough for the operator to estimate surgical margin. After the sample was resected, we dissected the specimen and got a tumor fluorescence imaging in vitro to assess the fluorescence intensity between tumor central and peripheral margin. The average fluorescence intensity of the tumor tissue, peritumor tissue, and normal tissue on the hand-held NIF spectrometer was 213.68 ± 34.98, 136.00 ± 17.31, and 101.40 ± 21.06 AUS, respectively (*P* < 0.05, *t* test) (Fig. 4). The NIF spectrometer was 105 ± 3.06 of DCIS, 107 ± 2.05 of mucinous adenocarcinoma, and 205 ± 13.06 of micropapillary carcinoma. The SBR of the tumor to the peritumor tissue and normal tissue was 1.54 ± 0.20 and 2.14 ± 0.60, respectively. The SBR was 2.45 ± 0.07 of DCIS，2.32 ± 0.62 of mucinous adenocarcinoma，and 2.25 ± 0.52 of micropapillary carcinoma. The fluorescence intensity of the tumor tissue was significantly greater than that of the peritumor tissue and normal tissue (*P* < 0.05, *t* test). Moreover, 3 had positive margins (6.98%) and 40 had negative margins (93.02%) according to the frozen pathology. Once the tumor was removed under the guidance of NIF imaging, the residual tumor tissue on the tumor bed was re-examined using the NIF imaging system. Abnormal ICG fluorescence was detected in 11.6% patients (5/43). Tissues showing abnormal fluorescence performed intraoperative complementary resection and pathologically examined. Finally, 3 patients were confirmed to have residual infiltrating carcinoma (Fig. 5a), whereas fluorescent cells in 2 patients were diagnosed as breast adenosis with ductal dilatation (Fig. 5b). At the fluorescence boundary, the sensitivity and specificity of NIF in distinguishing between normal and abnormal tissues were 93.3% and 96.0%, respectively.

## Discussion

At present, the NIF technique is being used to guide surgical resection of malignant tumors in the liver and gastrointestinal tract [14, 15] and has shown promising results in sentinel lymph node biopsies of breast cancer [16]. However, few studies on the use of ICG NIF imaging technology to determine the safest surgical margin in breast-conserving surgery for early breast cancer have been published to date.

The purpose of this study was to evaluate the ability of NIF to detect tumors and the feasibility of accurately judging the state of the incisal margin during breast-conserving surgery for breast cancer. In this study, 43 breast cancer patients tested positive for ICG. On color level imaging, the edge of an ICG fluorescence tumor was observed. During the operation, a NIF imaging system was used to detect tumor fluorescence. Pathological examination confirmed that residual cancer cells were not present in 40 cases (40/43) following tumor resection. The findings of this study demonstrated that a NIF imaging system based on ICG molecules is a feasible and effective method for determining the scope of resection and judging the state of the surgical margin during breast-conserving surgery for breast cancer. Accurate tumor edge recognition and radical tumor resection are required for the treatment of a variety of tumors, including breast cancer [17, 18]. At present, intraoperative margin evaluation techniques mainly include histology (frozen section and cytology) and imaging techniques (specimen X-ray, intraoperative ultrasound, and spectroscopy) for tumor margin evaluation. However, these techniques are insufficient to accurately predict the state of the tumor’s surgical margin and guide the operation [19]. Intraoperative ultrasound localization is one of the traditional methods. For patients with a large breast volume, it is difficult to distinguish between microcalcification and the depth difference from the skin incision to the main lesions. Air enters after the skin incision is made during the operation, which has a great influence when ultrasonic localization is needed for reconfirmation [20–22]. Rapid frozen sectioning is the most rapid and effective method to obtain the surgical margin during the operation. Intraoperative freezing evaluation is useful not only to judge the surgical margin relatively accurately but also to reduce additional tissue resection, thus improving the quality of life of patients after operation. Pathologists generally believe that frozen breast margins are difficult to describe. On the one hand, fat-containing tissue is difficult to freeze; on the other hand, it is an additional challenge to identify a small number of tumor cells from a large area of fat-containing tissue. Therefore, intraoperative freezing also has many limitations. Inaccurate results from frozen sections will affect the operation performed by surgeons, and false negatives from frozen margins will also be encountered in the clinic. For example, intraoperative freezing indicates that the margin is negative, whereas postoperative pathology suggests the presence of residual tumor in the margin [23, 24]. The high sensitivity of spectral-level imaging enables the detection of potentially malignant lesions in a new and feasible manner. In this study, the surgical margin was monitored in real time using spectral level imaging and fluorescence imaging. Spectral-level imaging is advantageous for detecting microfluorescence changes between cancer cells and normal tissues. The above studies established the supplementary value of NIF fluorescence imaging during radical resection of the tumor. Quantitative analysis of the ICG fluorescence intensity can be used to distinguish between tumor and normal tissues. The injection time and dose of ICG are critical for obtaining a clear tumor fluorescence image under the guidance of NIF imaging. A low dose reduces the tumor fluorescence, whereas a higher dose exceeds the metabolic capacity of normal tissue, blurring the boundary between the tumor and the surrounding normal tissue.

In summary, a near-infrared imaging system combined with ICG fluorescence molecular imaging not only has application value in the assessment of the development of primary breast cancer but also has the potential to reduce the positive rate of surgical margins and normal tissue damage, achieve accurate breast cancer resection, and provide a basis for noninvasive and efficient early diagnosis of breast cancer. However, since the small sample size of this study may lead to limitations and statistical uncertainty in the research conclusion, statistical software G*Power (Version 3.1.9.7, Heinrich-Heine-Universität, Düsseldorf, Germany) [25, 26] will be used to determine the sample size in subsequent studies to improve the statistical certainty. Its high sensitivity allows for the detection of small residual tumors following resection of the primary focus and is projected to become an auxiliary tool for establishing the surgical margins. Its future development will focus on the combination of the NIF contrast agent ICG and specifically targeted markers.

## Supplementary Information

**Additional file 1.**

## Data Availability

The datasets used or analyzed during the current study are available from the corresponding author on reasonable request.

## Abbreviations

BCS: Breast-conserving surgery
ICG: Indocyanine green
CFDA: China Food and Drug Administration
EPR: Enhanced permeability and retention effect
NIF: Near-infrared fluorescence
SBR: Signal-to-background ratio
DCIS: Ductal carcinoma in situ
